# Reproductive neuroendocrine defects programmed by prenatal testosterone treatment between gestational days 60–90 are amplified by postnatal obesity in sheep

**DOI:** 10.3389/fphys.2024.1436954

**Published:** 2024-08-02

**Authors:** S. C. Gurule, J. F. Sustaita-Monroe, L. N. King, R. S. Landers, V. Garza, S. M. West, S. E. Bynum, L. Perry, V. Padmanabhan, R. C. Cardoso

**Affiliations:** ^1^ Department of Animal Science, Texas A&M University, College Station, TX, United States; ^2^ Department of Pediatrics, University of Michigan, Ann Arbor, MI, United States

**Keywords:** androgens, neuroendocrine, obesity, PCOS (polycystic ovary syndrome), sheep

## Abstract

Polycystic ovary syndrome (PCOS) is the leading cause of anovulatory infertility in women of reproductive age, and obesity can increase the severity and development of the PCOS phenotype. Prenatal testosterone (T) treatment between gestational days 30–90 advanced puberty and disrupted the reproductive and metabolic phenotype in female sheep, recapitulating attributes of women with PCOS, with postnatal obesity amplifying its severity. On the other hand, prenatal T treatment from gestational days 60–90 led to a much milder phenotype. We hypothesized that reproductive neuroendocrine defects programmed by prenatal T treatment between gestational days 60–90 are amplified by postnatal obesity in sheep. Suffolk ewes received T propionate (T; 100 mg) or corn oil (C; vehicle) twice weekly from gestational days 60–90. At 5 months of age, T lambs were assigned to either a maintenance (100% of NRC requirements) or overfed (130% NRC) diet and C lambs were fed the maintenance diet. We compared the timing of puberty (n = 15/group) determined by twice weekly measurement of progesterone concentrations, estradiol positive feedback responsiveness (n = 8/group) determined by assessing LH secretion in response to exogenous estradiol, periovulatory LH dynamics during the second breeding season (n = 8/group) following synchronization with two injections of PGF2α, and progesterone negative feedback (n = 8/group) determined by characterizing LH pulses during the mid-luteal phase between C, T-maintenance and T-overfed groups. Our findings indicate that postnatal obesity: 1) exacerbated reproductive defects and further deteriorated reproductive cyclicity during the second breeding season (adulthood); 2) did not amplify the impairment in estradiol positive feedback in delaying the timing and amplitude of the LH surge, although it reduced the total amount of LH secreted during the preovulatory LH surge; 3) amplified the reduced responsiveness to progesterone negative feedback manifested as an increase in LH pulse amplitude and peak. These observations, in addition to supporting our previous findings that prenatal T treatment results in reproductive neuroendocrine dysfunction and periovulatory disruptions, provide evidence that these neuroendocrine defects programmed between gestational days 60–90 are amplified by postnatal obesity in female sheep.

## 1 Introduction

Polycystic ovary syndrome (PCOS) is the most common infertility disorder affecting up to 100 million women worldwide and approximately five million within the United States (Azziz et al., 2004). This complex syndrome is a multifactorial disease influenced by environmental factors on a predisposed genetic background (Legro et al., 1998) that modify metabolic and endocrine processes. PCOS is characterized by functional hyperandrogenism, polycystic ovaries, and anovulatory infertility. Obesity plays a significant role in the development and severity of the PCOS phenotype, as prevalence rates increase in obese women (Álvarez-Blasco et al., 2006; Yildiz et al., 2008). Prenatally testosterone (T)-treated sheep exhibit reproductive and metabolic perturbations that recapitulate those seen in women with PCOS (Padmanabhan and Veiga-Lopez, 2013).

The neuroendocrine system is impacted by prenatal T treatment in female sheep, resulting in neuroendocrine dysfunction. Disruptions in the three steroid feedback systems that regulate reproductive cyclicity include reduced responsiveness to estradiol negative feedback (Sarma et al., 2005), estradiol positive feedback (Sharma et al., 2002), and progesterone negative feedback (Robinson et al., 1999). In ruminants, decreased estradiol negative feedback and subsequent increase in luteinizing hormone (LH) pulse frequency can advance sexual maturation in females (Kinder et al., 1995). Defects in estradiol positive feedback mechanisms may alter significant features of the preovulatory LH surge, including the timing and amplitude of the surge, consequently impairing ovulatory capacity (Sharma et al., 2002). Progesterone negative feedback alterations result in an increase in LH pulse frequency during the luteal phase, which contributes to the development of functional hyperandrogenism and, ultimately, the persistence of follicles (Veiga-Lopez et al., 2008). Hypersecretion of LH and an imbalance of the LH:FSH ratio result in inadequate FSH concentrations, contributing to impaired follicle development. Of translational relevance, hyperandrogenic girls exhibit elevated pulsatile secretion of LH before puberty, indicating that these neuroendocrine defects likely develop early in the life of PCOS women (Apter et al., 1994). Animal models serve as exceptional assets to better understand the molecular and cellular mechanisms that underlie neuroendocrine dysfunction associated with hyperandrogenic conditions.

While the effects of prenatal T treatment for gestational days 30–90 on reproductive and metabolic function have been extensively characterized in female sheep, this study investigated the effects of postnatal obesity amplifying reproductive neuroendocrine defects programmed by T excess within a shorter gestation window of gestational days 60–90. Female sheep prenatally treated with T from gestational days 60–90 are phenotypically female (Wood and Foster, 1998) and show progressive loss of cyclicity, although at a significantly slower pace than female sheep prenatally treated with T between gestational days 30–90 (Birch et al., 2003). Since gestational day 60–90 females are not virilized, unlike gestational day 30–90 ewes, they serve as valuable resources for multigenerational studies. Identifying the susceptibility window in which neuroendocrine defects are programmed by T and amplified by postnatal obesity can be translationally relevant for preventing reproductive deficits associated with gestational hyperandrogenism in humans and may provide insights into the effects of obesity on PCOS development and severity.

This study was conducted to test the hypothesis that reproductive neuroendocrine defects programmed by prenatal T treatment during gestational days 60–90 are amplified by postnatal obesity in sheep. We hypothesized that: 1) postnatal obesity would advance puberty and amplify reproductive cycle defects in prenatal T-treated animals; 2) postnatal obesity would amplify the defects induced by prenatal exposure to T excess from days 60–90 of gestation on the estradiol-induced LH surge; 3) postnatal excessive weight gain would amplify the impact of prenatal T on parameters of the preovulatory LH surge and luteal defects; 4) the T-exposed maintenance-fed ewes would demonstrate a reduced responsiveness of the neuroendocrine axis to progesterone negative feedback during the mid-luteal phase of the estrous cycle compared to control females, and this effect would be further pronounced in T overfed ewes.

## 2 Materials and methods

All animal experimental procedures were approved by the Texas A&M University Institutional Animal Care and Use Committee (AUP 2019-0430).

### 2.1 Generation of experimental animals

All animals were housed at the Texas A&M University Physiology Field Laboratory facility. Suffolk (F_0_) ewes were bred via natural mating, and pregnancies were confirmed via transrectal ultrasonography on gestational days 30 and 60. Pregnant ewes received 100 mg of T propionate (Sigma-Aldrich Corp., St. Louis, MO) in 2 mL of corn oil via intramuscular injection twice weekly from days 60–90 of gestation (term = 147 days). Controls received an equal volume of corn oil (vehicle). Importantly, the circulating concentrations of androgens in the female fetuses achieved with this gestational T treatment are similar to the physiological ranges observed in the male offspring (Veiga-Lopez et al., 2011; Abi Salloum et al., 2015). Postnatally, the very low circulating concentrations of androgens typically observed in sheep have prevented us in previous studies from comparing circulating androgens between control and prenatal T-treated females (Manikkam et al., 2006). Ewe lambs were weaned at approximately 3 months of age and given a 2-month acclimation period before being assigned to nutritional treatments at approximately 5 months of age. Testosterone lambs were thereafter assigned randomly to gain either 0.3 kg/d (T maintenance: TM) or 0.4 kg/d (T overfed: TO). In any instance of T-treated twins, one lamb was assigned to the maintenance and the other to the overfed diet. Control (C) lambs were fed to gain 0.3 kg/d. Maintenance diet was designed to promote optimal growth without excess fat deposition, meeting the National Research Council (NRC) recommendations. Overfed females were provided a ration equal to 130% of the NRC recommendations, designed to achieve a body weight approximately 30% above that of the maintenance group. Ewe lambs were fed a complete lamb grower ration containing 16% crude protein, 3% crude fat, and 3% crude fiber twice daily. Body weights were collected bi-weekly beginning at weaning, and feed amounts were adjusted according to average daily gain requirements. Animals had *ad libitum* access to water and mineral supplementation. Lambs were regularly treated with anthelmintics to reduce parasitic infestations.

### 2.2 Experiment 1: impact of postnatal obesity on puberty and reproductive defects induced by prenatal testosterone excess for days 60–90 of gestation

This experiment was conducted during the first breeding season to determine the effects of T excess for gestational days 60–90 and postnatal body weight gain on 1) puberty attainment and 2) regularity of progesterone cycles during the first breeding season in female sheep. Blood samples were obtained via jugular venipuncture using serum vacutainer tubes to determine the time of puberty and to characterize the progesterone cycles of F_1_ generation ewe lambs. A subset of 15 ewe lambs were randomly selected from each treatment group of control, T maintenance, and T overfed ewe lambs. Concentrations of progesterone were measured in blood samples collected twice weekly from before the expected start of the breeding season until after the breeding season (August-January). Serum samples were stored at −20 °C, and progesterone concentrations were assessed via radioimmunoassay to evaluate average progesterone concentration, peak progesterone concentration, and cycle duration. The first occurrence of four continuous sample periods with progesterone concentrations less than 0.5 ng/mL, followed by three continuous sample periods greater than or equal to 0.5 ng/mL, was considered the date of puberty.

### 2.3 Experiment 2: impact of postnatal obesity on estradiol positive feedback defects induced by prenatal testosterone excess for days 60–90 of gestation

Blood samples were collected from 39 ewes (C, n = 14; TM, n = 12; TO, n = 13) twice weekly for 2 weeks prior to insertion of estradiol implants to confirm that ewes were in anestrous via progesterone radioimmunoassay. Seasonal anestrous was confirmed in all ewes by the absence of any blood sample with a progesterone concentration greater than 0.5 ng/mL, indicative of the absence of a functional corpus luteum. Eight ewes from each treatment group that had reached puberty (determined in experiment 1) and were confirmed to be in seasonal anestrous were randomly selected for this experiment. Each ewe received four 3 cm silastic estradiol implants, subcutaneously placed in the axillary area. Estradiol implants were placed using a previously described procedure to result in physiological, late-follicular phase estradiol concentrations (∼20 pg/mL) (Karsch and Foster, 1975; Kosut et al., 1997; Sharma et al., 2002). Blood samples were collected via jugular venipuncture every 2 h beginning 4 h prior to until 60 h after insertion of estradiol implants. Estradiol implants were removed immediately after sampling. Serum samples were stored at −20 °C until hormonal determinations. Estradiol concentrations were evaluated at hours −4, −2, 2, 6, 10, 20, 40, and 60 relative to insertion of estradiol implants. An estradiol-induced LH surge was defined if LH concentrations increased for at least three consecutive samples (6 h) above twice the average concentration secreted prior to insertion of the estradiol implants (Herbosa and Foster, 1996).

### 2.4 Experiment 3: impact of postnatal obesity on periovulatory hormonal imbalances induced by prenatal testosterone excess for days 60–90 of gestation

**FIGURE 1:**
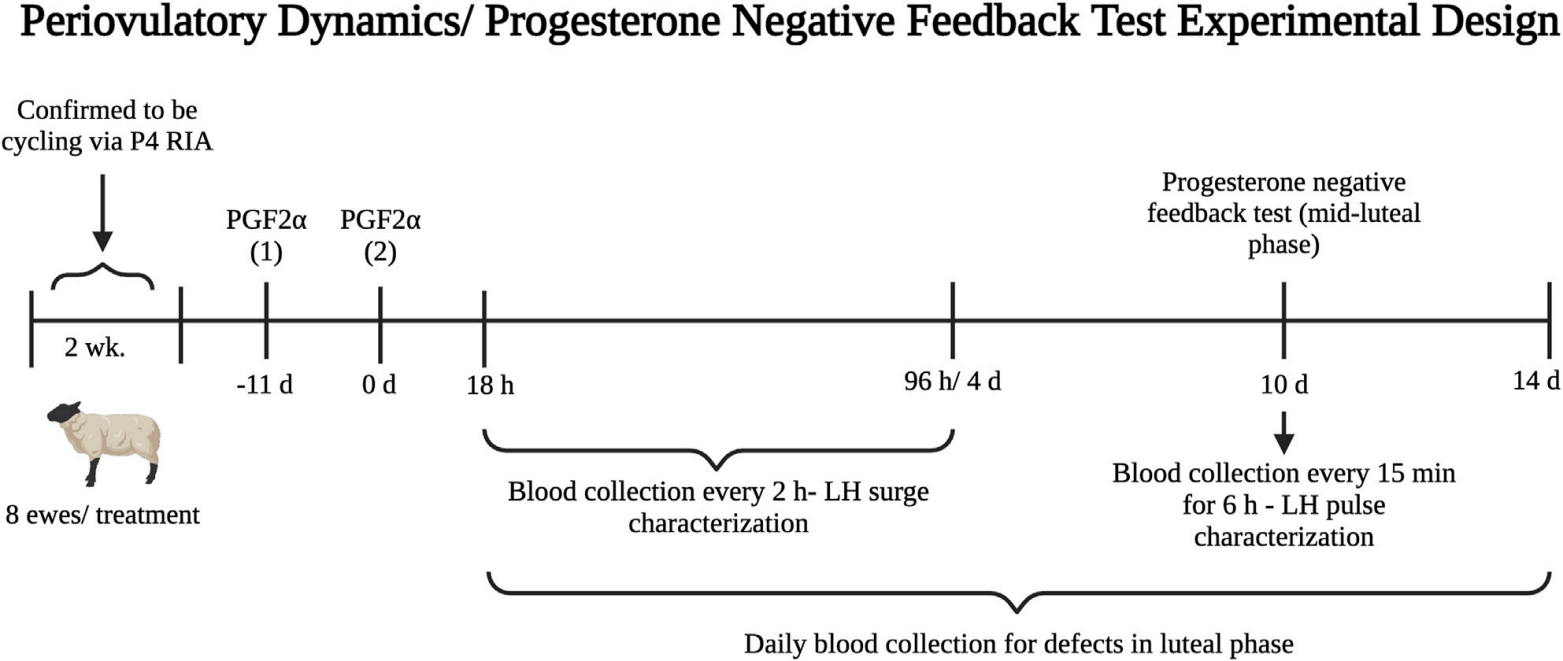
Experimental design timeline for the periovulatory dynamics (experiment 3) and progesterone negative feedback (experiment 4) studies performed during the second breeding season (early adulthood).

This experiment investigated the activational role of postnatal obesity in amplifying the effects of T excess from gestational days 60–90 in disrupting the preovulatory LH surge in sheep. Blood samples were collected twice weekly for 2 weeks prior to the start of experiment 3 to confirm that ewes were ovulating via progesterone radioimmunoassay. Ewes with at least two consecutive samples with progesterone concentrations greater than or equal to 0.5 ng/mL were considered cycling. Eight ewes from each treatment group that were confirmed to be cycling were randomly selected to receive prostaglandin F_2α_ injections. For estrus synchronization, each ewe received two doses of 20 mg prostaglandin F_2α_ (Lutalyse, Zoetis, Kalamazoo, MI) via intramuscular injection 11 days apart. Blood samples were collected via jugular venipuncture using heparin vacutainer tubes every 2 h beginning 18 h after the second PGF_2α_ injection until 96 h to assess the LH surge (Figure 1). Plasma samples were stored at −20 °C until hormonal determinations. An endogenous LH surge was considered present if LH concentrations for at least six consecutive samples (10 h) were greater than twice the mean concentration of the baseline (first six samples spanning hours 18–28 after the second PGF_2α_ injection) (Sharma et al., 2002).

### 2.5 Experiment 4: impact of postnatal obesity on progesterone negative feedback defects induced by prenatal testosterone excess for days 60–90 of gestation

This experiment investigated the contribution of postnatal overfeeding in amplifying the effects of T treatment for gestational days 60–90 in disrupting the responsiveness of the neuroendocrine axis to the progesterone negative feedback in sheep. The same 24 ewes from experiment 3 (n = 8/group) were used in experiment 4. Ten days after the second PGF_2α_ injection was administered, progesterone negative feedback sensitivity was assessed during the mid-luteal phase. Blood samples were collected via jugular venipuncture every 15 min for 6 h to characterize LH pulse secretion. Additionally, blood was collected daily via jugular venipuncture beginning on the morning of the second PGF_2α_ injection (day 1) for 2 weeks to assess progesterone secretion during the luteal phase (Figure 1). During sampling, ewes had *ad libitum* access to water, minerals, and hay. Ewes remained on normal feeding routine to minimize induced stress.

For the progesterone negative feedback test, ewes that met the following criteria were selected for LH pulse characterization: 1) produced a LH surge in response to prostaglandin F_2α_ synchronization (assessed in experiment 3); 2) progesterone concentration was greater than 1 ng/mL on day 10 after the second PGF_2α_ injection (mid-luteal phase); 3) had two daily samples proceeding and following day 10 that had greater than or equal to 0.5 ng/mL of progesterone. Pulses of LH were defined based on the following criteria: 1) a secretory episode composed of two consecutive samples exhibiting a concentration greater than the mean concentration of the two previous samples; 2) maximum concentration greater than the nadir value of the two points that preceded it by more than two standard deviations of this nadir (Viguie et al., 1995).

### 2.6 Radioimmunoassays

Circulating concentrations of progesterone were determined in duplicates using a commercial radioimmunoassay kit (Coat-A-Count, MP Biomedicals, Solon, OH) with modifications for ovine serum (standards were prepared via serial dilution of progesterone using serum from ovariectomized ewes). Sensitivity of the progesterone assay averaged 0.17 ng/mL with an average intra- and inter-assay coefficient of variation (CV) of 10.7% and 7.6%, respectively. A previously validated LH double antibody radioimmunoassay (McVey Jr and Williams, 1991; Amstalden et al., 2003) was used to measure circulating concentrations of LH to characterize the endogenous and estradiol-induced LH surge, as well as LH pulses during the progesterone negative feedback test. Samples were assayed in duplicate and an ovine LH-specific primary antibody was used (graciously provided by Dr. A.F. Parlow; AFP-192279) The sensitivity of the LH assay averaged 0.14 ng/mL, with an average intra- and inter-assay CV of 7.7% and 11.4%, respectively. Circulating concentrations of estradiol were measured in duplicate samples via a double antibody radioimmunoassay, adapted from Kirby et al. (1997). Estradiol radioimmunoassay was performed on samples from all animals of the estradiol positive feedback test to confirm that estradiol implants were producing appropriate concentrations and to dismiss any complications with estradiol implants. The sensitivity of the estradiol assay averaged 0.67 pg/mL, with an average intra- and inter-assay CV of 6.3% and 8.1%, respectively. Duplicates with a CV greater than 15% and binding below assay sensitivity (∼80% binding) were re-assayed for all radioimmunoassays.

### 2.7 Statistical analyses

JMP software (SAS Institute, Inc.) was used for statistical analyses. For all analyses, differences were considered significant when *p* < 0.05 and a tendency when *p* ≤ 0.10 and ≥ 0.05. Comparison of age at puberty, average progesterone concentration, peak progesterone concentration, and cycle duration among treatment groups were performed using ANOVA with Tukey HSD *post hoc* analysis. A comparison of onset, duration, amplitude, peak, and area under the curve (AUC) of the LH surge among treatment groups was performed using ANOVA with Dunnet’s *post hoc* analysis. Chi-squared analysis was used to compare the proportion of ewes responding to the stimulatory feedback action of estradiol with a produced LH surge. A comparison of onset, duration, amplitude, peak, and AUC of the LH surge among treatment groups was performed using ANOVA with Dunnet’s *post hoc* analysis. Chi-squared analysis was used to compare the proportion of ewes cycling at the beginning of the breeding season and those responding to PGF_2α_ by exhibiting an LH surge. Pulse frequency, amplitude, and peak, as well as mean LH concentration were compared among treatment groups using ANOVA with Dunnet’s *post hoc* analysis.

## 3 Results

### 3.1 Onset of puberty and reproductive cyclicity

**FIGURE 2:**
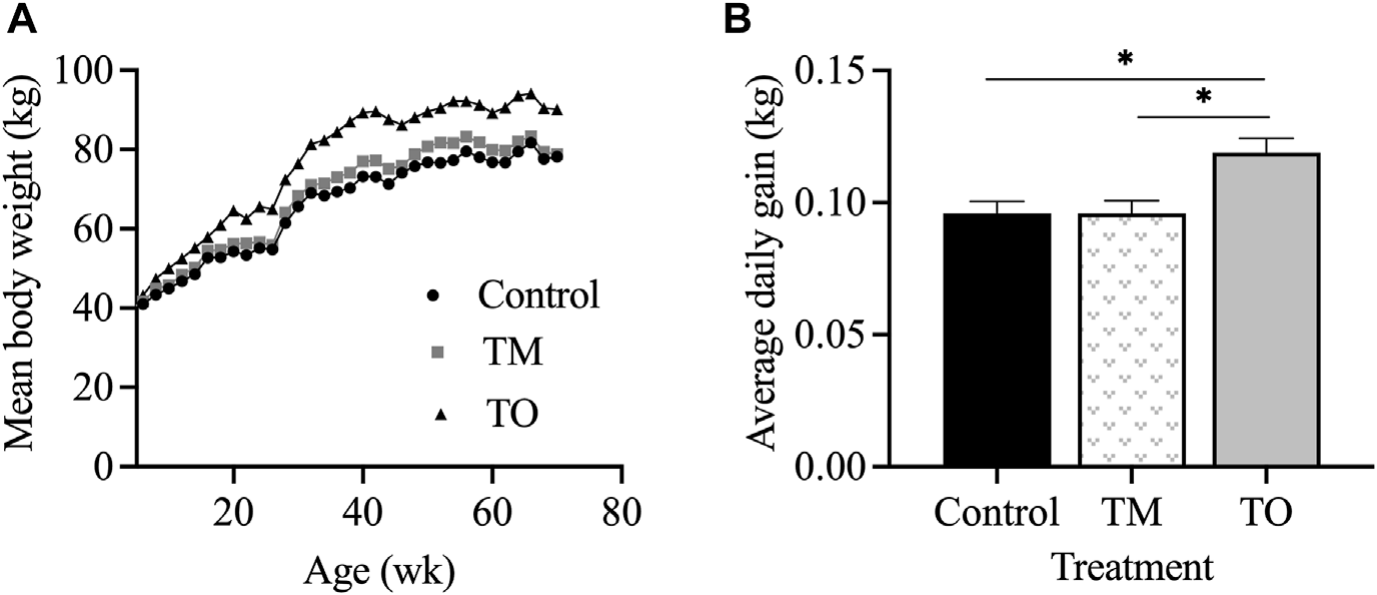
**(A)** Mean body weight and **(B)** average daily gain for ewes in the control, testosterone maintenance (TM) and testosterone overfed (TO) treatment groups from time of weaning (5 weeks) until the end of experiment 4 (∼70 weeks of age at time of the progesterone negative feedback test).

**FIGURE 3:**
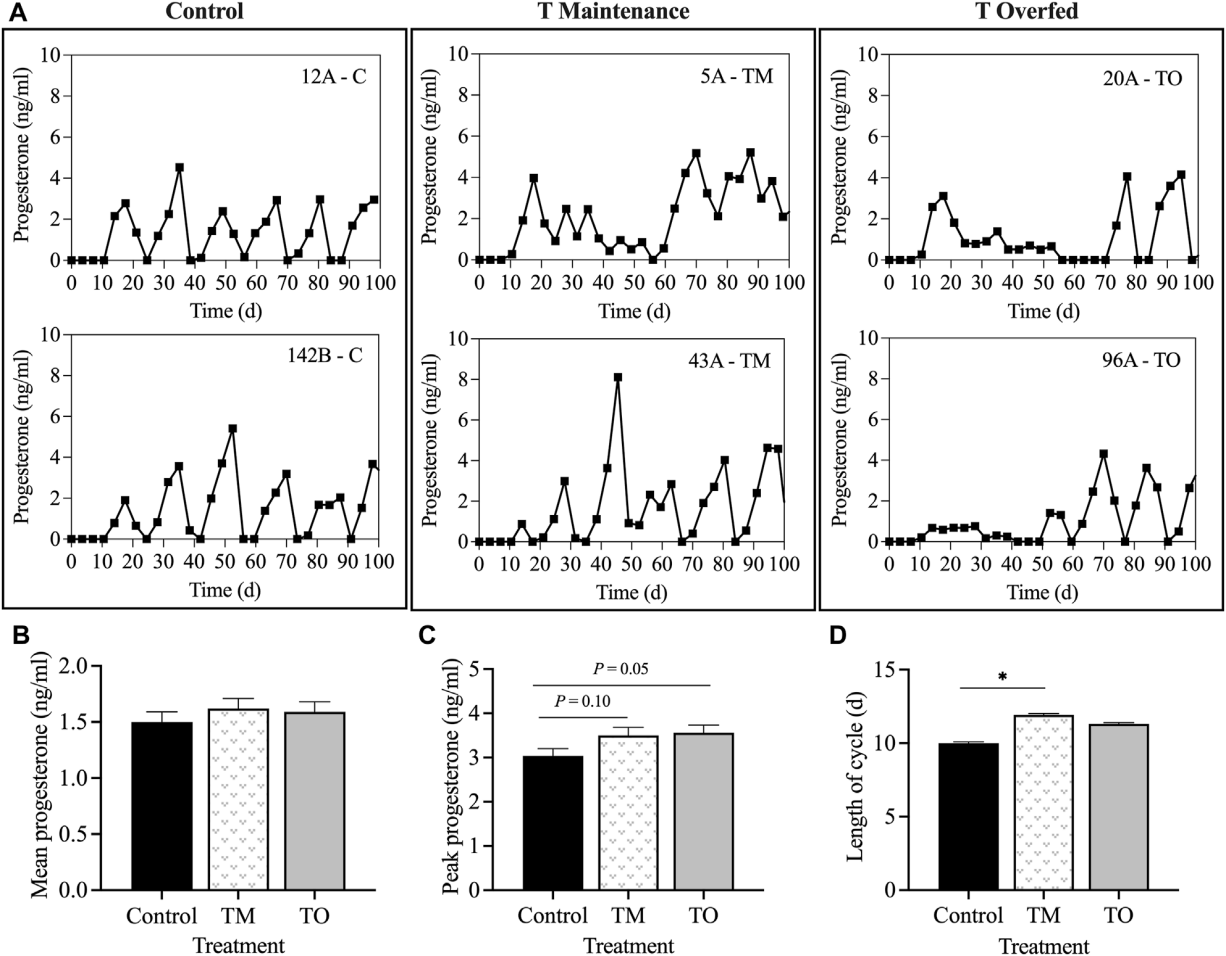
**(A)** Two representative progesterone profiles of control, testosterone maintenance (TM), and testosterone overfed (TO) females from first ovulation until the end of the first breeding season. **(B)** mean ± SEM progesterone concentrations during the first breeding season. **(C)** mean ± SEM peak progesterone concentration during the first breeding season. **(D)** mean ± SEM length of luteal phase of the estrous cycle between the three treatment groups throughout the first breeding season. *p < 0.05.

Figure 2 demonstrates that the rate of body weight gain of ewes followed the proposed nutritional design for this study because a significant difference in body weight (*p* < 0.01) and average daily gain (*p* < 0.05) was apparent between maintenance fed (C and TM) and overfed (TO) groups at experimental time points when estradiol positive feedback, progesterone negative feedback, and periovulatory dynamics sampling were performed. Average daily gain from the time of weaning until the beginning of the first breeding season did not differ across treatments (C = 0.23 ± 0.02 kg; TM = 0.25 ± 0.01 kg; and TO = 0.27 ± 0.02 kg). Six animals (C, n = 1; TM, n = 3; TO, n = 2) did not attain puberty during the first breeding season and were subsequently assigned the last day of the breeding season as date of first ovulation. When comparing body weight at puberty, no difference was observed across treatments (C = 50.33 ± 2.42 kg; TM = 52.58 ± 2.42 kg; and TO = 57.39 ± 2.42 kg). Age at puberty also did not differ between groups (C = 274.6 ± 9.9 days; TM = 270.0 ± 11.3 days; and TO = 266.0 ± 10.2 days). Figure 3 depicts the progesterone cycles from two representative animals from each group beginning at the onset of puberty through the first breeding season. In control animals, progesterone cycles followed a normal pattern, where the corpus luteum formed and regressed continuously throughout the breeding season. Testosterone maintenance and overfed ewe lambs demonstrated cycle defects by persisting in the luteal phase for a long time and/or temporarily or permanently ceasing to have progesterone cycles. Average progesterone concentration was not different between groups. Peak progesterone concentration was greater (*p* = 0.05) for TO and tended (*p* = 0.10) to be greater for TM than for controls. Cycle duration was greater (*p* < 0.05) for TM lambs than for controls. However, cycle length was not different between the TO group and the C and TM groups (Figure 3). Additionally, no differences were observed for the number of cycles exhibited during the first breeding season between treatment groups.

### 3.2 Responsiveness of the neuroendocrine axis to estradiol positive feedback

Average circulating estradiol concentration prior to estradiol implant insertion was not different when compared between treatment groups (C = 0 pg/mL; TM = 0.37 ± 0.34 pg/mL; and TO = 0.57 ± 0.34 pg/mL). However, average estradiol concentration after implant insertion was greater (*p* < 0.05) in TO ewes (22.86 ± 1.37 pg/mL) than in C (17.04 ± 1.37 pg/mL) and greater (*p* < 0.0001) compared to TM ewes (11.87 ± 1.27 pg/mL). Additionally, average estradiol concentration throughout the entire sampling period was greater (*p* < 0.01) in TO (20.13 ± 2.03 pg/mL) than in TM (9.58 ± 2.03 pg/mL) but not C females (14.71 ± 1.60 pg/mL).

**FIGURE 4:**
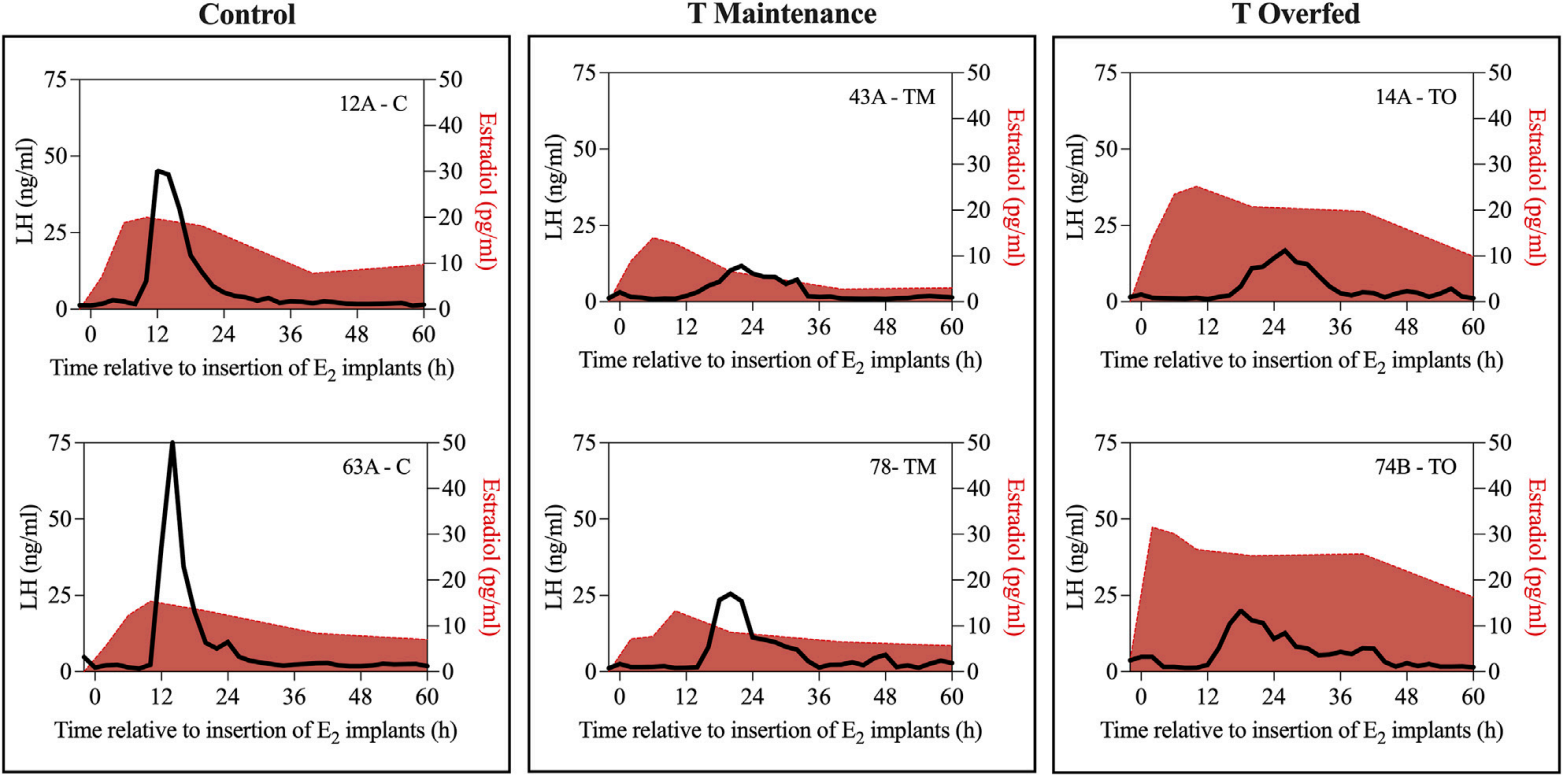
Representative profiles of two ewes from each treatment group (Control [C], T maintenance [TM], and T overfed [TO]) during the estradiol-induced LH surge. Luteinizing hormone surge is indicated by the black line and estradiol concentration is represented by the red shading.

**FIGURE 5:**
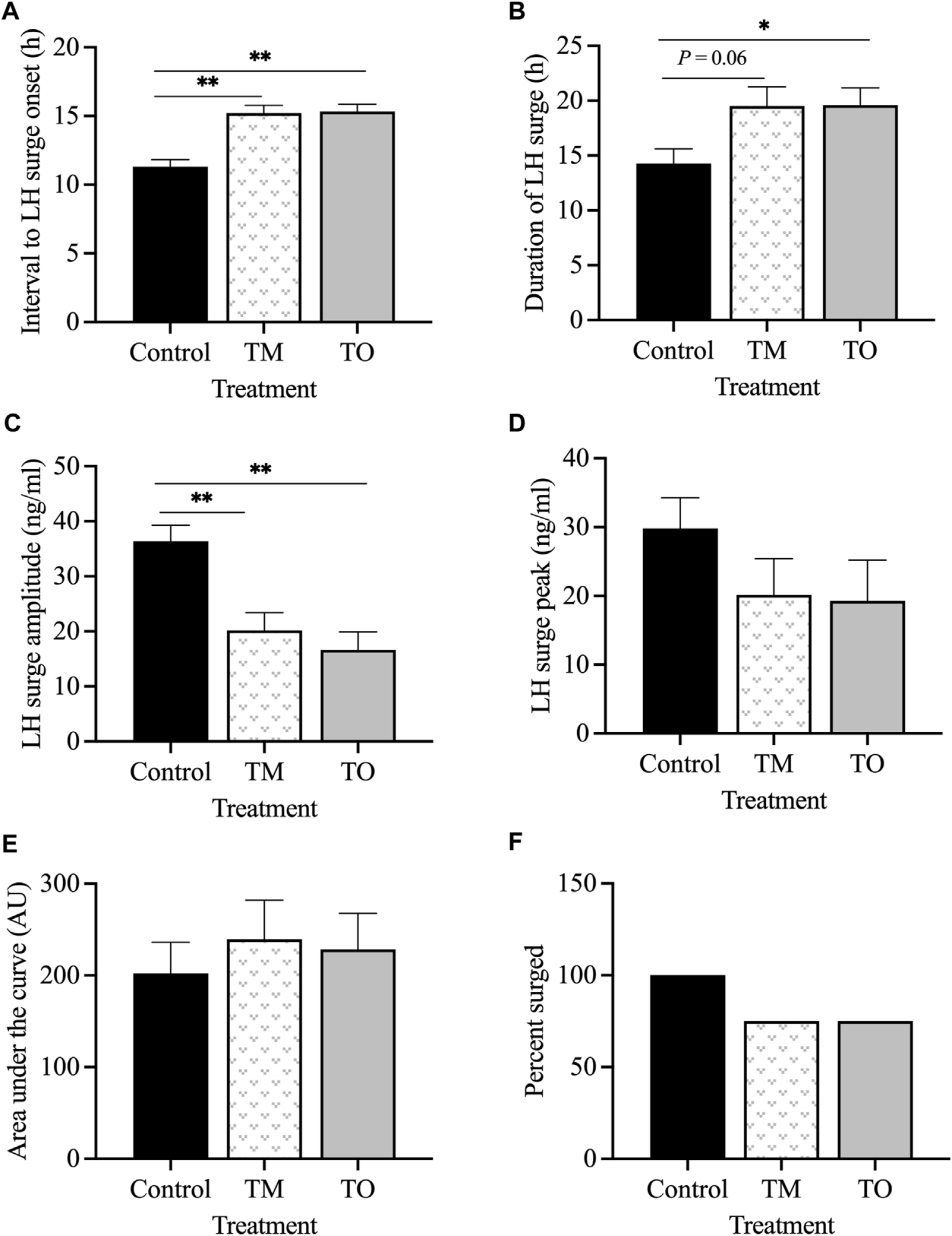
**(A)** Mean ± SEM interval between the insertion of E_2_ implants and onset of the LH surge; **(B)** mean ± SEM duration of the E_2_-induced LH surge; **(C)** mean ± SEM amplitude of the E_2_-induced LH surge; **(D)** mean ± SEM peak of the E_2_-induced LH surge; **(E)** mean ± SEM area under the curve (AUC) of the E_2_-induced LH surge; **(F)** percentage of estradiol-induced LH surge response between the three treatment groups during the anestrous period. *p < 0.05; **p < 0.01. C: control; TM: testosterone maintenance; TO: testosterone overfed.

Figure 4 depicts the representative profiles of two ewes from each treatment group during the estradiol-induced LH surge. Onset of the LH surge in relationship to the time of insertion of the E_2_ implants was delayed (*p* < 0.05) in both the TM and TO groups compared to the controls (Figure 5A). Duration of the LH surge was greater (*p* < 0.01) in the TO and tended (*p* = 0.06) to be greater in TM females compared to the controls (Figure 5B). Luteinizing hormone surge amplitude was greater (*p* < 0.01) in the C ewes compared to both the TM and TO females (Figure 5C). Peak concentration (Figure 5D) and the AUC (Figure 5E) of the LH surge were not different between the three treatment groups. Additionally, the percentage of estradiol-induced LH surge response was not different between the three treatment groups during the anestrous period (Figure 5F).

### 3.3 Periovulatory hormonal dynamics

At the beginning of the second breeding season, all control (17/17; 100%), 9/18 (50%) TM, and 9/18 (50%) TO ewes were confirmed to be cycling via progesterone radioimmunoassay. Chi-square analysis indicated a difference (*p* < 0.01) between treatment groups, with fewer TM and TO ewes cycling at the beginning of the breeding season compared to controls.

**FIGURE 6:**
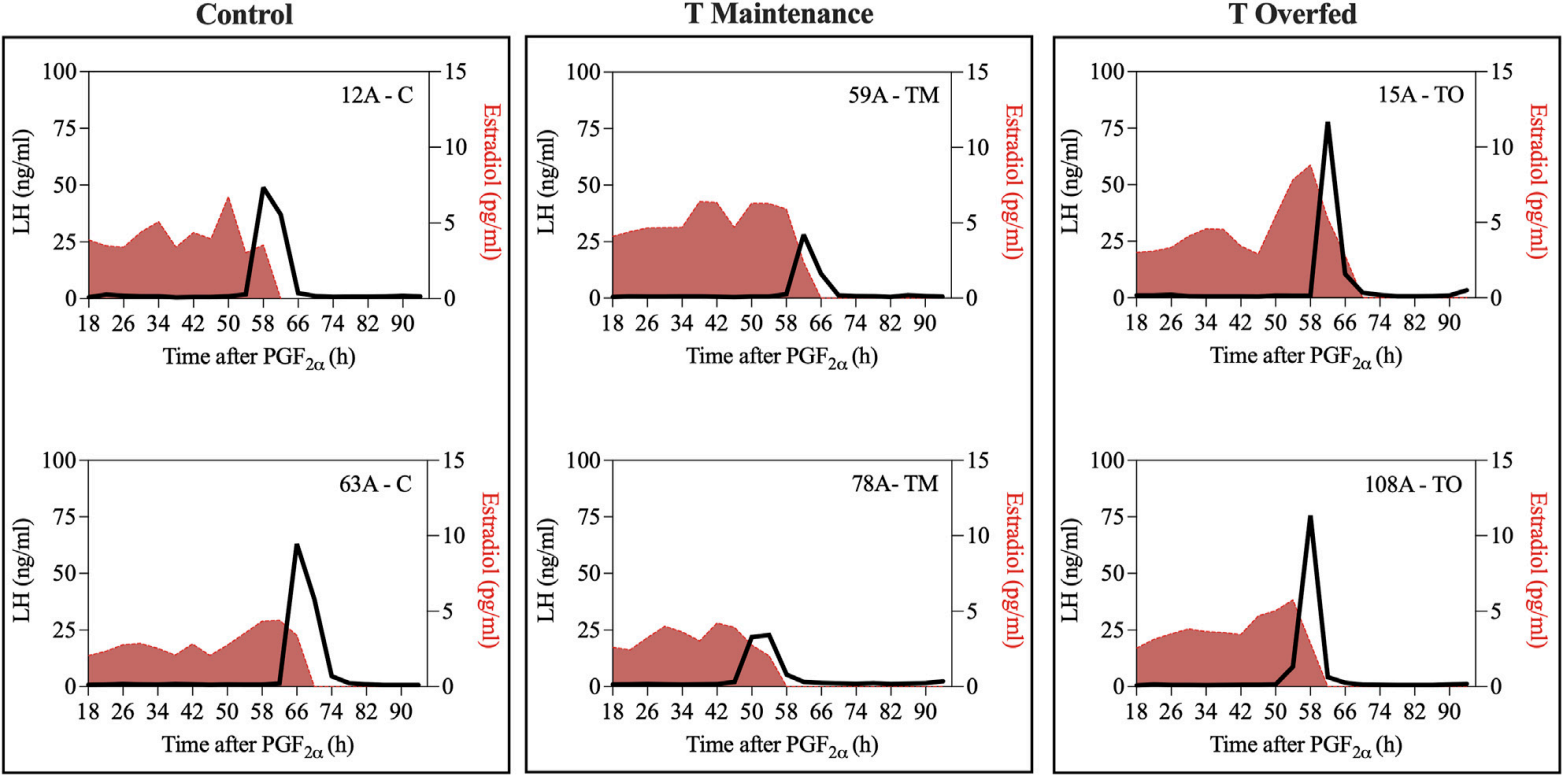
Two representative profiles of each treatment group (Control [C], T maintenance [TM], and T overfed [TO]) during the preovulatory LH surge. Luteinizing hormone concentrations are indicated by the black line and estradiol concentrations are shaded in red.

**FIGURE 7:**
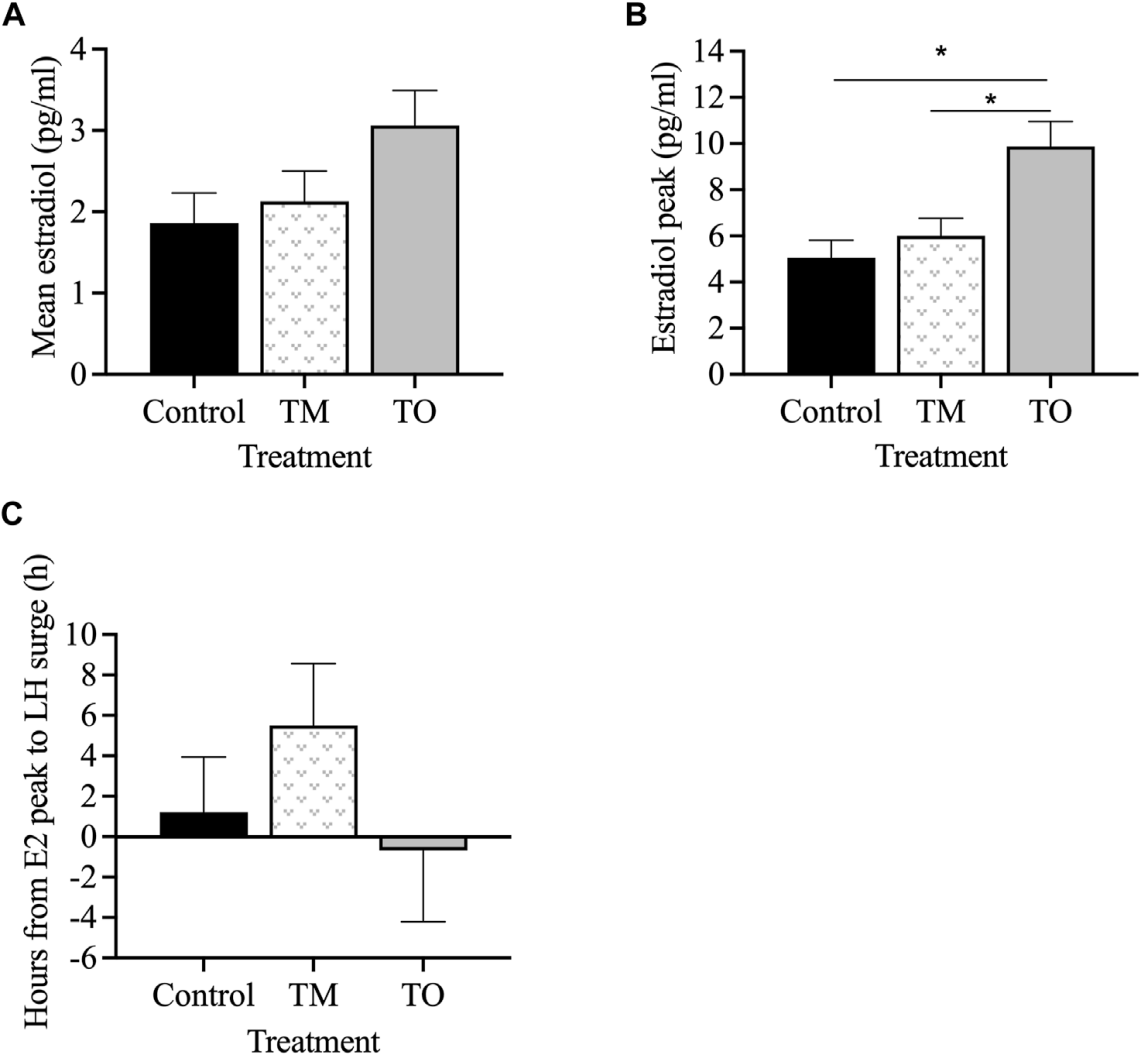
**(A)** Mean estradiol concentrations, **(B)** average peak estradiol concentrations, and **(C)** interval between estradiol peak and onset of the LH surge between the three treatment groups (Control [C], T maintenance [TM], and T overfed [TO]) during the preovulatory surge during the second breeding season. *p < 0.05.

Figure 6 depicts the profiles of estradiol and LH concentrations during the periovulatory period for two representative animals for each group. Mean estradiol concentrations throughout the periovulatory period ranged between 2 and 3 pg/mL and were not different between treatment groups (Figure 7A). However, mean estradiol peak concentration was greater (*p* < 0.05) in TO ewes compared to TM and C ewes (Figure 7B). The interval between the estradiol peak and the onset of the LH surge was not different between the three treatment groups (Figure 7C).

**FIGURE 8:**
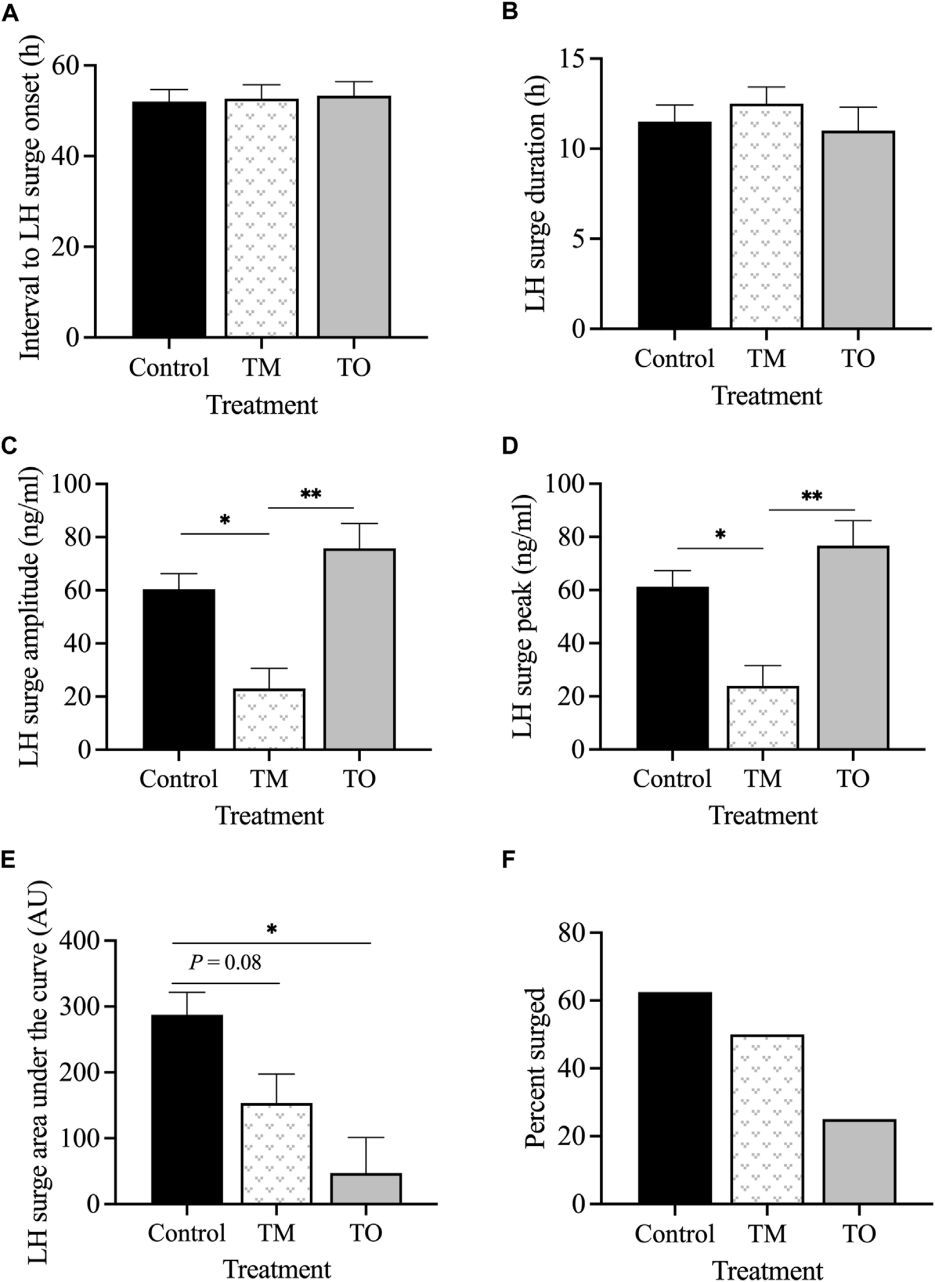
**(A)** Mean interval between the second PGF_2α_ injection and the onset of the LH surge; **(B)** mean duration of the LH surge; **(C)** mean amplitude of the LH surge; **(D)** mean peak concentration of the LH surge; **(E)** total LH secreted during the LH surge (area under the curve); **(F)** percentage of PGF_2α_-induced LH surge response between the three treatment groups during the second breeding season (early adulthood). Treatment groups (Control [C], T maintenance [TM], and T overfed [TO]). *p < 0.05; **p < 0.01.

The interval between the second PGF_2α_ injection and the onset of the LH surge (Figure 8A) and the LH surge duration (Figure 8B) did not differ between groups. However, the LH surge amplitude was lower (*p* < 0.05) in TM, but not TO, than for C ewes (Figure 8C). Peak concentration of the LH surge was also lower (*p* < 0.05) in TM but not TO, compared to C ewes (Figure 8D). The LH surge AUC, a proxy of total LH secreted, was lower (*p* < 0.05) in TO and tended (*p* = 0.08) to be lower in TM ewes compared to controls (Figure 8E). The percentage of ewes that exhibited a LH surge in response to the two PGF_2α_ injections did not differ among treatment groups (Figure 8F).

Progesterone concentrations throughout the entire sampling period were not different between the three treatment groups when normalized to time at onset of the LH surge (C = 3.03 ± 0.40 ng/mL; TM = 3.73 ± 0.44 ng/mL; TO = 3.39 ± 0.51 ng/mL). In addition, progesterone concentration for the first 5 days post LH surge did not differ between groups (C = 0.43 ± 0.13 ng/mL; TM = 0.60 ± 0.15 ng/mL; TO = 0.39 ± 0.17 ng/mL). Mean progesterone peak concentration was also not different among the three treatment groups (C = 7.54 ± 0.95 ng/mL; TM = 9.39 ± 1.07 ng/mL; TO = 8.34 ± 1.23 ng/mL).

### 3.4 Responsiveness of the neuroendocrine axis to progesterone negative feedback

**FIGURE 9:**
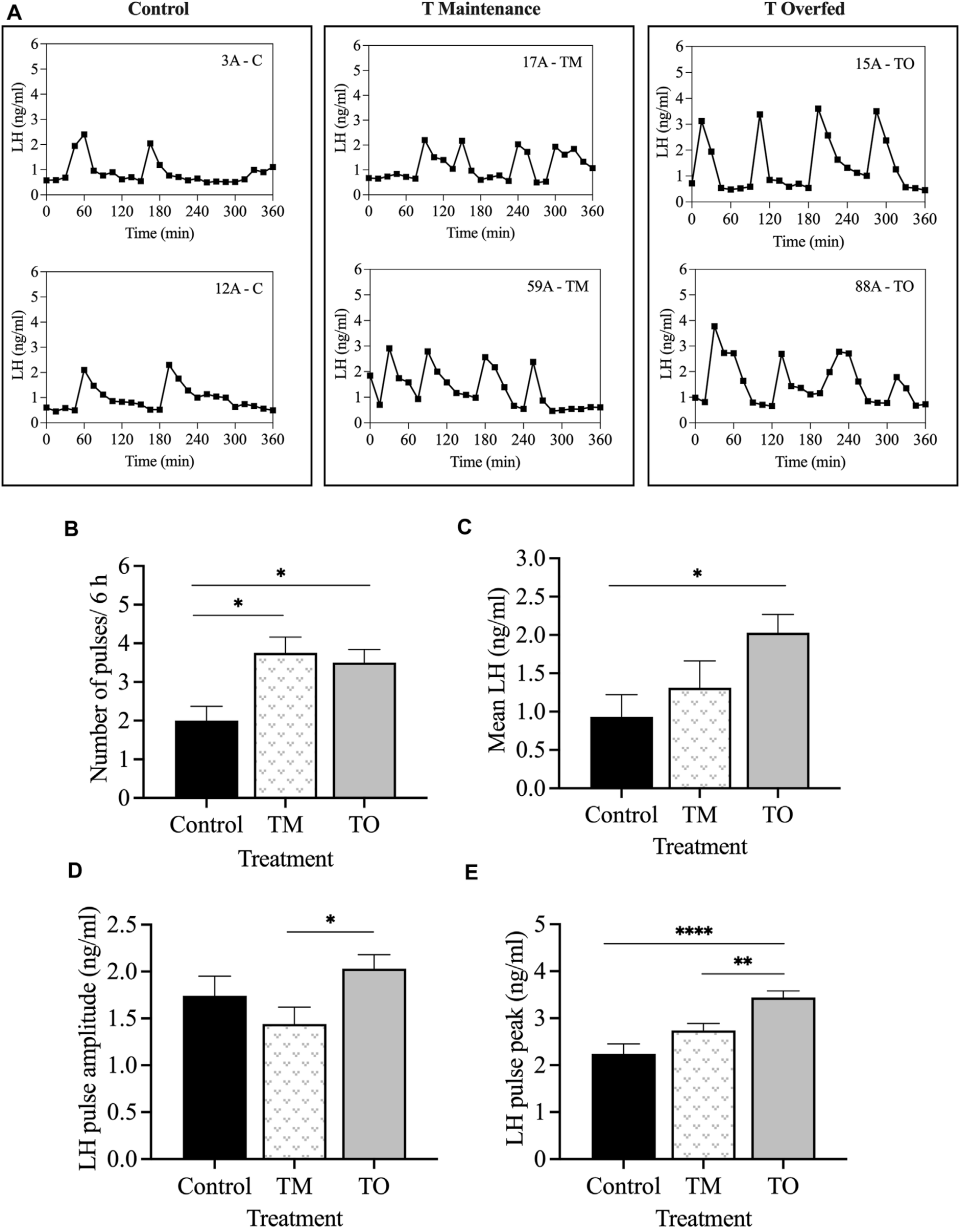
**(A)** Representative LH pulse profiles of two animals from each of the three treatment groups (Control [C], T maintenance [TM], T overfed [TO]) during the progesterone negative feedback test, conducted during the mid-luteal phase; **(B)** mean ± SEM number of LH pulses within the 6 h sampling period; **(C)** mean ± SEM LH concentration; **(D)** mean ± SEM LH pulse amplitude concentration; **(E)** mean ± SEM LH pulse peak concentration between the three treatment groups during the progesterone negative feedback test. *p < 0.05; **p < 0.01; ***p < 0.001.

The mean concentrations of progesterone were not different among the groups on day 10 after the second PGF_2α_ injection (mid-luteal phase), when the progesterone negative feedback test was carried out. Figure 9A depicts the LH profiles during the 6-h sampling period for two representative animals for each group. The frequency of LH pulses was greater (*p* < 0.05) in TM and TO ewes compared to C females (Figure 9B). Additionally, the mean LH concentration during the 6-h sampling period was greater (*p* < 0.05) in TO, but not TM, compared to controls (Figure 9C). The mean LH pulse amplitude concentration was greater (*p* < 0.05) in TO compared to TM, but not to C ewes (Figure 9D). Moreover, the mean LH pulse peak concentration was greater (*p* < 0.001) in TO compared to controls and greater (*p* < 0.01) compared to TM ewes (Figure 9E).

## 4 Discussion

Using an animal model of PCOS-like phenotype, our findings, in addition to supporting our previous observations that prenatal T treatment results in reproductive neuroendocrine dysfunction, demonstrate that these neuroendocrine defects programmed between gestational days 60–90 are amplified by postnatal obesity in female sheep. More specifically, our findings demonstrate that postnatal excessive weight gain exacerbates the reproductive defects seen during early adulthood and further deteriorates reproductive cyclicity, reducing the total amount of LH secreted during the preovulatory LH surge, and amplifying the reduced responsiveness to progesterone negative feedback manifested as an increase in LH pulse frequency, amplitude, and peak. Translationally, these results may provide insights into the effects of obesity on the severity of PCOS pathogenesis in women. The interpretation of each neuroendocrine feature tested and their potential relevance to women with PCOS and other hyperandrogenic conditions are discussed below.

### 4.1 Onset of puberty and reproductive cyclicity

Ewe lambs treated with T from gestational days 30–90 have previously been shown to exhibit advanced puberty attainment compared to control animals (Kosut et al., 1997; Wood and Foster, 1998; Puttabyatappa et al., 2016). However, in the present study (gestational days 60–90 model), age at puberty did not differ between control and T treated ewe lambs, suggesting that the programming of early puberty occurs within the 30–60 gestational window. This finding is corroborated by earlier studies (Short, 1974; Clarke et al., 1976; Wood et al., 1991; Wood et al., 1995) demonstrating that the critical period for sexual differentiation of the surge system occurs during gestational days 30–90 in female sheep. In adolescent girls, childhood obesity has been shown to be associated with advanced puberty, increased menstrual cycle irregularities, and PCOS (Itriyeva, 2022). In the gestational day 30–90 T-treated and postnatal overfed (25% above maintenance) sheep model, Steckler et al. (2009) also demonstrated no difference in time of puberty but observed that T overfed females stopped cycling earlier during the first breeding season (December 10 ± 21.8 days) compared to overfed controls (February 23 ± 11.4 days) and T normal-fed females (April 25 ± 44.4 days); additionally, overfeeding resulted in a tendency for lower mean peak progesterone concentrations. Modest weight loss of 5%–10% has been shown to improve ovulatory function in women with PCOS (Clark et al., 1998; Huber-Buchholz et al., 1999; Crosignani et al., 2003; Moran et al., 2008), indicating the impact of obesity on normal reproductive function.

As demonstrated in the present study, reproductive progesterone cycles were disrupted in T maintenance ewes during the first breeding season and further amplified in T overfed ewes. Alterations in which the corpus luteum demonstrated pronounced delay in regression or prolonged corpus luteum lifespan throughout the first breeding season suggest an early indication of reproductive failure and future fertility issues. Previously, gestational T exposure from gestational days 30–90 decreased the number of cycles and increased the duration of progesterone cycles during the first breeding season in Suffolk ewes (Manikkam et al., 2006). Subluteal cycle occurrences were higher in T treated (6/14) than in control ewes (0/16) during the first breeding season (Manikkam et al., 2006). Additionally, Manikkam et al. (2006) reported a reduced proportion of ewes with normal cycles in the first breeding season of 64% to only 25% in the second breeding season. Similarly, Unsworth et al. (2005) reported that only 64% of gestational day 30–90 T-treated ewes showed reproductive cycles. Similar to our results, previous studies reported that prenatal T treatment between gestational days 60–90 T resulted in fewer regular reproductive cycles (Birch et al., 2003) and a shorter breeding season (Birch et al., 2003; Steckler et al., 2009). Abnormalities in reproductive cycles associated with perinatal T excess also occur in other species such as rodents (Gorski, 1968) and primates, showing prolonged intervals between menstrual cycles followed by a greater occurrence of luteal phase defects (Goy and Robinson, 1982). In the current study, overfeeding moderately normalized cycle duration during the first breeding season in T females. However, previous studies with a longer T treatment window (30–90) found that postnatal excess weight gain amplifies the adverse effects of prenatal T excess on reproductive function (Steckler et al., 2009). While the basis for the discrepancy between the present study and Steckler et al. (2009) is not clear, it is possible that the short interval between the beginning of the nutritional interventions and the first breeding season (postpubertal age) was not sufficient to induce adverse effects on reproductive function in this study. As discussed later in experiments 3 and 4, clear detrimental effects of postnatal overnutrition on reproductive function were observed at later time points during the second breeding season (early adulthood) in these animals.

### 4.2 Responsiveness of the neuroendocrine axis to estradiol positive feedback

Based on previous studies, the estradiol-induced LH surge was predicted to be delayed and key characteristics altered due to prenatal T treatment. In the present study, both groups of T treated ewes showed reduced responsiveness to estradiol positive feedback, demonstrated by the delayed onset of the LH surge. Duration of the surge was longer in T overfed females and tended to be longer in T maintenance animals compared to controls. Additionally, a key feature observed to be altered in this estradiol positive feedback test was reduced amplitude of the LH surge in T overfed ewes and T maintenance ewes compared to controls. The delay in onset of the LH surge in both prenatal T treated groups may have detrimental effects on oocyte competence (Freeman et al., 1970) and a successful pregnancy, as an ovulatory delay is correlated with a higher incidence of chromosomal abnormalities (Witschi and Laguens, 1963). A delay in onset of the LH surge of 24–48 h in mice (Butcher and Fugo, 1967; Skinner et al., 2000) and 11 h in cattle (van de Leemput et al., 2001) results in a decreased ovulation rate and comprised follicular development (D'Occhio et al., 1999; Carrière et al., 2002). Although differences in LH surge characteristics were seen in prenatal T groups, there appears to be no significant amplification due to postnatal excessive weight gain.

Similarly, in a gestational day 60–90 ovary-intact sheep model, onset of the LH surge was delayed 6 hours compared to controls (Sharma et al., 2002). The delay was even longer in gestational day 30–90 T treated ewes, with a 12 h delay compared to the controls (Sharma et al., 2002). The timing of LH/FSH surge peaks was delayed in gestational days 30–90 T-treated ewes but not in dihydrotestosterone (DHT)-treated females compared to controls, supporting the idea that estradiol positive feedback disruptions are not programmed by T directly but rather by the conversion of T into estradiol. However, since DHT can be converted to 3β-androstendiol and act through ERβ, other studies have raised the possibility that both androgens and estrogens synergize in programming the estradiol feedback in prenatal T treated sheep (Padmanabhan et al., 2015; Puttabyatappa et al., 2016). Additionally, these studies demonstrated that estradiol positive feedback disruptions were progressive, as onset of the LH surge was more delayed during the pubertal period than at 12 weeks of age, indicating continued postnatal organization of the surge system (Veiga-Lopez et al., 2009). While the lower amplitude LH surges observed in prenatal T-treated sheep are likely sufficient to induce the ovulation of a dominant follicle, they may not be sufficient to support the development of a fully-functional corpus luteum that is able to produce high amounts of progesterone to support pregnancy (Robinson et al., 2005).

Although estradiol implants were designed to secrete physiological estradiol concentrations to be similar between the three treatment groups, circulating concentrations of estradiol were significantly greater in the T overfed group. This may be due to the well-known expression of aromatase and estrogen production in the adipose tissue (Grodin et al., 1973). Aromatase is regulated by FSH in the ovary, but some prominent controllers in adipose tissue are glucocorticoids, class 1 cytokines, tumor necrosis factor alpha (TNFα) (Simpson, 2003), and leptin (Brown et al., 2009). The increase in adiposity and a presumed increase in leptin secreted from adipocytes in the T overfed ewes could be a possible reason for the increase in circulating estradiol concentrations seen in our study. A positive correlation between estradiol and leptin is seen in PCOS patients, regardless of their weight (Mendonça et al., 2004). Increased leptin concentration in PCOS women (Micić et al., 1997; Rizk and Sharif, 2015) can interact with the coexistence of obesity and altered glucose tolerance seen in PCOS. A hyperestrogenic state is promoted by PCOS, demonstrated by a clinical study where women with PCOS had higher estradiol levels which led to more severe hyperandrogenism than control women (Chen et al., 2015). Although estradiol concentrations were significantly greater in T overfed females, that was not sufficient to prevent a reduction in the mean LH surge amplitude, further suggesting disruptions at the neuroendocrine level.

### 4.3 Periovulatory hormonal dynamics

At the beginning of the breeding season, circulating progesterone concentrations indicated progressive reproductive cycle deterioration in both T treated groups (50% cyclicity in each group) compared to 100% cyclicity in control ewes. Notably, all ewes used in this experiment were the same as those confirmed as pubertal in the first breeding season (experiment 1). These data are consistent with previous studies and further demonstrate the premature reproductive senescence observed in female sheep prenatally treated with T.

The preovulatory estradiol peak was greater in both T treated groups compared to control ewes. A possible reason for this may be persistent follicles from the ovary generating an increase in estradiol production. Previous research has indicated an increase in androgen receptor (AR) expression in granulosa cells of prenatal T-treated ewes that may result in increased AR signaling and subsequent follicular persistence (Ortega et al., 2009). Furthermore, hyperinsulinemia is commonly manifested in women with PCOS, and the prenatal T treated sheep used in the present study were found to be insulin resistant (Cardoso et al., unpublished observations), which may increase insulin-like growth factor (IGF1) expression. Insulin-like growth factor and hyperinsulinemia play an important role in ovarian function. The stimulatory effects of IGF1 on estrogen production in granulosa cells due to their ability to enhance gonadotropin action on ovarian follicular steroidogenesis has been well documented (Spicer et al., 2002). Similarly, prenatal T treated rhesus monkeys exhibited increased *IGF1* mRNA abundance in granulosa, theca, and in the interstitial compartment, as well as increased IGF1 receptor mRNA concentration in thecal cells (Vendola et al., 1999).

Luteinizing hormone is responsible for the reactivation of meiosis and oocyte maturity prior to ovulation (Dekel et al., 1990), and an inappropriate release of LH may have detrimental effects on the release and fertilization of the oocyte (Homburg et al., 1988). Similar to the poor surge response seen in the present study, previous studies reported that 100% of control ewes but only 71% (Veiga-Lopez et al., 2009) or 67% (Padmanabhan et al., 2015) of ewes treated with T between gestational days 30–90 exhibit an LH surge in response to PGF_2α_ administration. Veiga-Lopez et al. (2009) also observed an increase in LH surge magnitude during the natural cycle in all groups; in contrast, our study showed similar concentrations during both anestrous and breeding season periods. Time from the second PGF_2α_ injection to onset of the LH surge was not different among groups in the current study but has previously been reported to be delayed in gestational day 30–90 T-treated ewes (Steckler et al., 2009). In our study, LH surge amplitude and peak were reduced in T maintenance fed ewes compared to controls, consistent with Veiga-Lopez et al. (2009). Collectively, these findings indicate that some disruptions in the periovulatory surge release of LH—that is, a reduction in LH surge peak and amplitude—are programmed between gestational days 60–90, while other disruptions, such as a delay in the onset of the LH surge, are likely programmed earlier during fetal development between days 30–60 of gestation. Regarding the programming pathways, prenatal androgen antagonist cotreatment fully restored LH surge response to the level of the control group in prenatal T ewes treated from gestational days 30–90, suggesting that these alterations are programmed via the androgen signaling pathway (Padmanabhan et al., 2015). However, prenatal androgen antagonist cotreatment could not prevent the delay in onset of the LH surge and normalize amplitude of LH surges in prenatal T ewes, suggesting that a different pathway, such as aromatization of T into estradiol, may be involved (Padmanabhan et al., 2015). While postnatal obesity did not statistically decrease the percentage of ewes that exhibited a LH surge in response to PGF_2α_ administration (50% of TM ewes surged vs. 20% of TO ewes), excessive weight gain resulted in a significant reduction in total LH secretion during the surge (area under the curve) compared to control ewes, which could have further detrimental effects on release and fertilization of the oocyte as well as corpus luteum formation.

### 4.4 Responsiveness of the neuroendocrine axis to progesterone negative feedback

Robinson et al. (1999) showed that the suppression of LH pulsatile secretion by progesterone is sexually differentiated in sheep, as the ram is less sensitive to the progesterone negative feedback than the ewe. They demonstrated that prenatal androgen exposed ewes had disrupted response of both pulsatile and surge release of GnRH to progesterone negative feedback. Interestingly, this reduced responsiveness to the progesterone feedback mechanism was observed in female sheep prenatally treated with T during either gestational days 30–90 or 60–90, suggesting that the shorter gestational T treatment window of 60–90 days of gestation is sufficient to program this neuroendocrine defect. The findings from the present study corroborate this premise, with both T maintenance and T overfed groups exhibiting higher LH pulse frequency during the mid-luteal phase of the estrous cycle compared to controls. Interestingly, prenatal T-treated ewes postnatally overfed not only exhibited higher LH pulse frequency but also manifested an increase in mean LH pulse amplitude compared to prenatal T-treated ewes, which further amplifies the LH hypersecretion during the luteal phase of the estrous cycle. Differentiation in the amplitude of LH pulses may reflect either the sensitivity of the pituitary or the amount of LH stored there. One plausible reason for the increased LH pulse amplitude observed in prenatal T-treated overfed ewes may be due to their elevated energy status and a likely increased activation of metabolic signaling pathways within the anterior pituitary. Gonadotropes in the anterior pituitary express IGF-1R, the receptor for IGF-1 (Unger and Lange, 1997), and several studies have reported that IGF-1 stimulates LH synthesis and/or secretion (Soldani et al., 1994; Soldani et al., 1995; Xia et al., 2001). Moreover, gonadotropes also express the leptin receptor (Iqbal et al., 2000; Akhter et al., 2014), and *in vivo* and *in vitro* studies provide evidence that leptin modulates the expression and secretion of gonadotropins (De Biasi et al., 2001; Akhter et al., 2014; Odle et al., 2018). Therefore, in addition to the programming effects of prenatal T excess, postnatal excessive weight gain likely creates a metabolic platform that amplifies the LH hypersecretion in this sheep model of PCOS phenotype.

From a mechanistic standpoint, previous studies have shown that prenatal T treatment reduced the number of dynorphin and progesterone receptor-positive cells in the arcuate nucleus of the hypothalamus in female sheep without impairing kisspeptin expression (Cheng et al., 2010; Moore et al., 2021). Therefore, these results suggest that the decreased responsiveness to the progesterone negative feedback in prenatal T treated sheep may be due, at least in part, to stimulatory effects of kisspeptin expression outweighing the inhibitory effects of dynorphin, thus reducing the capacity of progesterone to inhibit GnRH secretion (Cheng et al., 2010).

From a clinical perspective, data suggest that androgen excess impairs progesterone’s ability to act within the hypothalamus and suppress GnRH secretion, therefore contributing to the development of LH hypersecretion in women with PCOS and other hyperandrogenic conditions (Ruddenklau and Campbell, 2019). This is supported by data demonstrating that PCOS women require higher amounts of progesterone to suppress LH secretion compared to levels of non-PCOS women (Pastor et al., 1998) and through evidence that long-term treatment with an androgen antagonist, flutamide, restores normal progesterone sensitivity in women with PCOS (Eagleson et al., 2000). Furthermore, PCOS individuals with high LH levels had a significantly higher prevalence of infertility (Conway et al., 1989), decreased chance of conception, and increased risk of miscarriage than PCOS women with normal LH concentrations (Homburg et al., 1988), highlighting the clinical importance of addressing the LH hypersecretion state. While studies investigating the effects of obesity on LH hypersecretion in PCOS women are scarce, our current results in a sheep model of PCOS phenotype suggest that excessive weight gain could further amplify this neuroendocrine defect.

## 5 Conclusion

In support of previous studies, postnatal overfeeding exacerbated the reproductive defects seen in prenatal T treated sheep, further deteriorating reproductive cyclicity during the second breeding season without impacting age at puberty. In addition, estradiol positive feedback was impaired in both T maintenance and T overfed females through a delayed LH surge and reduced LH surge amplitude, which can have detrimental effects on ovulatory capacity and oocyte competence. Key features of the preovulatory LH surge were impaired, with no further amplification of postnatal overfeeding observed. Lastly, progesterone negative feedback was reduced in prenatal T maintenance fed ewes and T overfed ewes through an observed increase of LH pulse frequency. Additionally, LH pulse amplitude and peak were amplified by postnatal overfeeding. Together, these studies demonstrate that prenatal exposure to androgen excess between gestational days 60–90 results in an array of reproductive neuroendocrine defects, some of which are further deteriorated by postnatal excessive weight gain. However, it is important to note that postnatal obesity can program reproductive neuroendocrine defects in the absence of prenatal insults. Since the present study design did not include a control overfed group, it is not possible to completely distinguish the effects of postnatal obesity amplifying the effects of prenatal T excess *versus* the effects of postnatal obesity by itself. Future studies including a control overfed group will be required to address this matter.

Fetal exposure to excess steroids during development and the consequent impact on adult health remains a major public health concern. Our data in female sheep support the idea that short-term exposure to T excess is sufficient to program the neuroendocrine axis resulting in reproductive defects and subsequent sub- or infertility during adulthood. Additionally, these results provide insight into the effects of postnatal obesity in amplifying periovulatory and neuroendocrine dysfunction. As childhood obesity prevalence continues to rise, metabolic imbalances may amplify prenatal insults, increasing the demand for intervention strategies to ameliorate the pathology. Due to experimental constraints on the use of human tissue for biomedical research, animal models such as prenatal T-treated sheep are unique assets to helping identify intervention strategies for prevention of development of PCOS phenotype, and/or treatment to ameliorate the severity and progression of the disease.

## Data Availability

The original contributions presented in the study are included in the article/Supplementary Material; further inquiries can be directed to the corresponding author.
